# Supplementary material for: Vampyromorph coleoid predation by an ichthyosaurian from the Early Jurassic Lagerstätte of Bascharage, Luxembourg
Source: PeerJ. 2025 Sep 8;13:e19786. doi: 10.7717/peerj.19786 (PMC12424614; doi:10.7717/peerj.19786)
Supplement: Supplemental Information 1 — The data stems from Maxwell (2012) and was complemented with MNHNL TV211. (A) PCoA analysis of the full dataset, original data with z-transform. (B), PCA of the dataset without pelvic elements, original data with z-transform. (C), PCoA of the full dataset, scaled data with z-transform. (D), P CA of the dataset without pelvic elements, scaled data with z-transform. [file peerj-13-19786-s001.pdf]

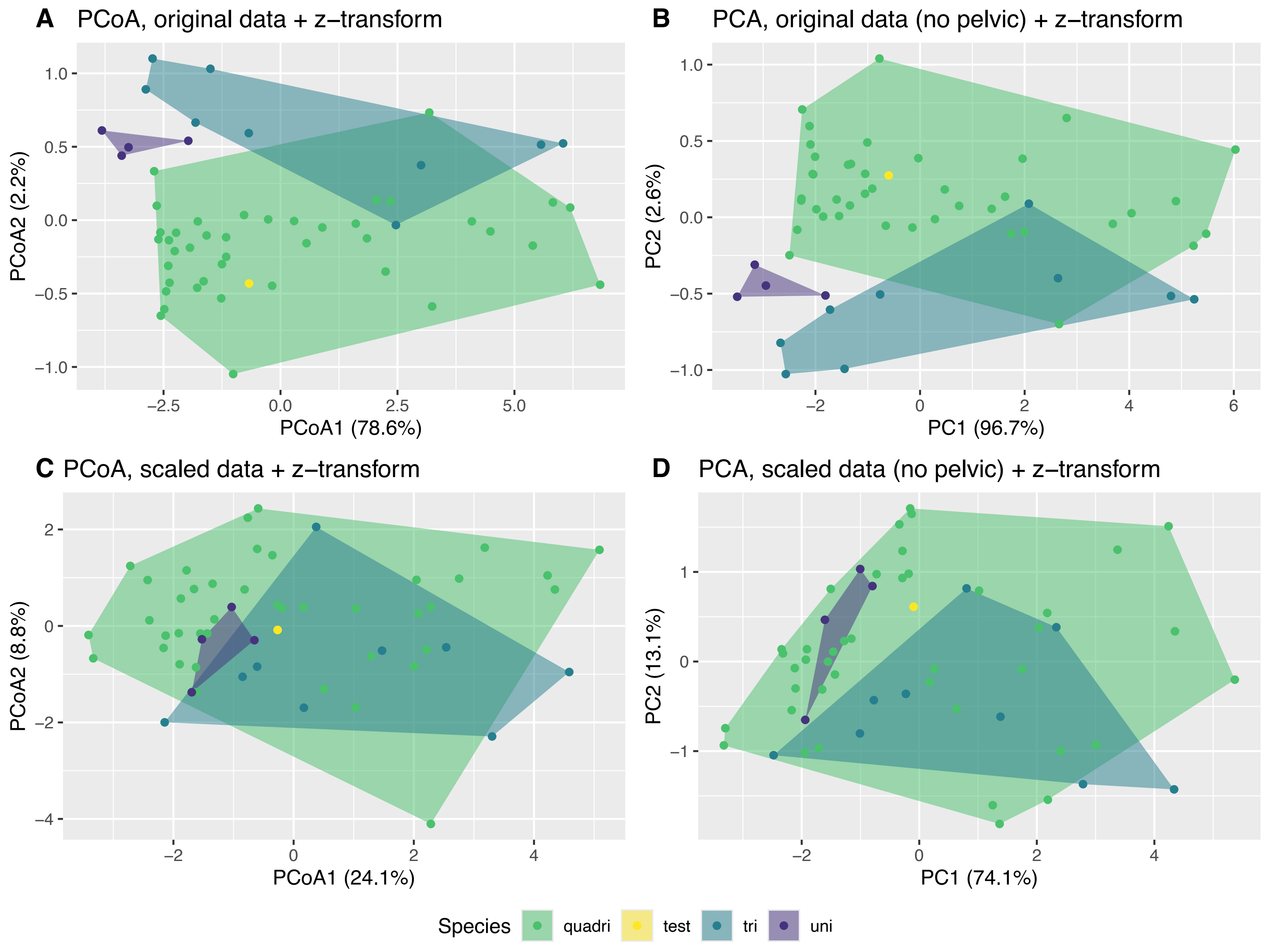

A PCoA, original data + z−transform
PCoA1 (78.6%)
PCoA2 (2.2%)
B PCA, original data (no pelvic) + z−transform
PC1 (96.7%)
PC2 (2.6%)
C PCoA, scaled data + z−transform
PCoA1 (24.1%)
PCoA2 (8.8%)
D PCA, scaled data (no pelvic) + z−transform
PC1 (74.1%)
PC2 (13.1%)
Species
quadri
test
tri
uni
